# Associations between sleep duration, sleep quality, and weight status in Chinese children and adolescents

**DOI:** 10.1186/s12889-022-13534-w

**Published:** 2022-06-07

**Authors:** Huan Chen, Li-Juan Wang, Fei Xin, Guo Liang, Yuan Chen

**Affiliations:** School of Physical Education and Sport Training, Shanghai University of Sport, Yangpu District, Changhai Road No 399, Shanghai, 200438 China

**Keywords:** Sleep, Adiposity, Child, Adolescence, Relationship, China

## Abstract

**Background and objective:**

The high prevalence of obesity is a serious problem, and sleep is considered to be a factor for obesity. This study aimed to examine the relationship between sleep duration, sleep quality, and weight status among children and adolescents in China and to explore whether the relationship between sleep duration and weight status is independent of sleep quality. Sex and age differences were also explored.

**Methods:**

A cross-sectional research was conducted among 2019 children and adolescents (1029 boys and 990 girls) aged 8–16 years in Shanghai. An open-question was used to obtain data on sleep duration, which was categorized into the following four groups based on the age-specific National Sleep Foundation Sleep Duration Recommendations: 1) very short, 2) short, 3) recommended, and 4) long. The Pittsburgh Sleep Quality Index was used to assess sleep quality. Weight and height were measured for all participants. The variable weight status was estimated with the Chinese children and adolescent age- and sex-specific body mass index (BMI) and was categorized into overweight/obesity and normal weight.

**Results:**

Short sleep duration (7–8 and 6–7 h for 6–13 and 14–16 years old, respectively) significantly increased odds of overweight/obesity (OR = 1.32, 95% CI: 1.06–1.64) compared with that of the recommended sleep duration (9–11 and 8–10 h for 6–13 and 14–16 years old, respectively). The relationship between the two variables existed independent of sleep quality. No significant relationship was found between sleep quality and overweight/obesity of children and adolescents. Sleep quality (OR = 1.07, 95% CI: 1.01–1.14) and short sleep duration (OR = 1.51, 95% CI: 1.06–2.13) increased the risk for overweight/obesity among girls, whereas no significant relationships between sleep duration, sleep quality, and overweight/obesity were found among boys. Short sleep duration increased the risk of overweight/obesity in children aged 8–13 years (OR = 1.34, 95% CI: 1.05–1.71), independent of sleep quality, but no significant relationships between these two variables existed for adolescents aged 14–16 years.

**Conclusions:**

Overall, short sleep duration increased the risks of overweight/obesity in children and adolescents in China, independent of sleep quality. This relationship is significant for girls and children aged 8–13 years instead of boys and adolescents aged 14–16 years. Interventions to extend the sleep duration of children and adolescents, especially girls and children aged 8–13 years in China, are necessary to improve their weight status.

**Supplementary Information:**

The online version contains supplementary material available at 10.1186/s12889-022-13534-w.

## Background

The prevalence of childhood overweight and obesity is an undeniable concern worldwide [1, 2]. China, as the largest developing country, experienced a rapid increase in obese and overweight school-aged children and adolescents in the past three decades. Compelling evidence from the largest nationally representative survey, namely, the Chinese National Survey on Students’ Constitution and Health (CNSSCH), demonstrated that the prevalence of overweight and obesity in children and adolescents aged 7–18 increased from 1.3% in 1985 to 19.4% in 2014 [3]. Moreover, overweight and obese levels were higher among boys (24.2%) than girls (14.6%) and decreased with age [3]. The high prevalence of overweight and obesity in Chinese children and adolescents is attributed to the change in traditional diet patterns, increased sedentary behavior, and decreased physical activity (PA) participation, with the economic transition and development since the 1970s [4]. The prevalence of pediatric obesity led to numerous physical health consequences (e.g., cardiovascular diseases, type 2 diabetes, and sleep apnea), mental and motor developmental delays, and psychological problems throughout the lifespan [5–7].

Numerous factors (e.g., PA, sedentary time, and dietary habits) were identified to be associated with children and adolescent obesity [8, 9]. Among these factors, the relationship between sleep duration, sleep quality, and obesity of children and adolescents was investigated in several studies, but inconsistent findings were achieved [10, 11]. The majority of studies found that short sleep duration increased the risk of obesity among children and adolescents [12–14], whereas a small number of studies found nonsignificant relationships between short sleep duration and obesity [15]. Considering sleep quality, some studies found that children and adolescents with poor sleep quality increased odds of gaining weight [10, 16], whereas no significant relationships between the two variables were found in other studies [17, 18]. Therefore, the relationships between sleep duration, sleep quality and obesity remain uncertain. Additional studies are necessary to shed more light on the relationship between sleep and weight status among children and adolescents, especially among children and adolescents in China wherein sleep pattern is different from Western countries and research evidence on the sleep–obesity relationship of children and adolescents is limited [19, 20].

Some researchers proposed that daily sleep duration and quality may vary between boys and girls [21–23]. Age increase may also make sleep duration and quality difference because of the change of sleep needs and patterns [24, 25]. Thus, sex and age differences may exist in the effect of sleep duration and quality on weight status [26, 27]. However, available evidence regarding the differential effect of sex and age on the association between sleep and the risk of obesity is limited and inconclusive [23, 28–30]. A meta-analysis on the sleep–obesity relationship of children and adolescents by Guidolin and Gradisar [31] emphasized a future research recommendation to investigate whether demographic factors (e.g., sex) interact with sleep and obesity. Moreover, further studying this association by sex and age is necessary due to the differences in the prevalence of obesity between boys and girls and between children and adolescents in China [3, 26].

Three research purposes are introduced in the present study considering the aforementioned discussion: (1) to investigate the association between sleep duration, sleep quality, and weight status among children and adolescents in China, (2) to explore whether the relationship between sleep duration and weight status of children and adolescents is independent of sleep quality, and (3) to examine sex and age [i.e., children aged 6–13 years and adolescents aged 14–16 years recommended by the U.S. National Sleep Foundation (NSF)] difference in the sleep–obesity relationship.

## Materials and methods

This cross-sectional study was reported in accordance with the Strengthening the Reporting of Observational Studies in Epidemiology Statement [32].

### Survey design and sample selection

**Fig. 1:**
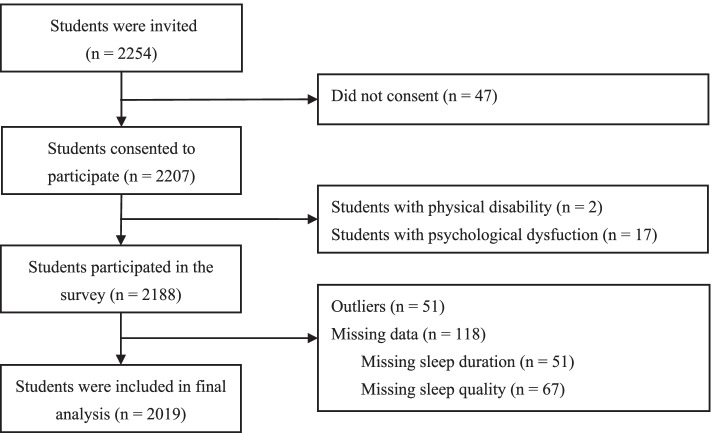
The procedure used for cleaning invalid and missing data in this study

A cross-sectional survey was conducted from October to December 2020. Multistage cluster random sampling was used to select participants from three elementary schools and three middle schools located in five districts in Shanghai, a city in the eastern part of China. The principals of these schools agreed to participate in this study. Elementary and secondary schools in Shanghai have five (i.e., grades 1–5 with students aged 6–11 years old) and four (i.e., grades 6–9 with students aged 12–16 years old) grades, respectively. Grade one and two students (aged 6 to 7) were excluded from this study because they are too young to understand and complete the survey accurately. Three to seven classes were randomly selected from each grade among grades three to nine, and all students in each class were invited to participate in the study. Accordingly, a total of 2254 students from 72 classes were invited to participate in the present study. Signed informed consent forms were sent to the students and their parents or guardians, and 2207 students volunteered to participate. Data from 2188 participants were included for further analysis after excluding those with any serious physical disability (*n* = 2) or psychological dysfunction (*n* = 17). An initial inspection of the raw data showed that 169 participants had either missing data on sleep duration (*n* = 51) and quality (*n* = 67) or provided data that were out of normal range (*n* = 51). Removal of the missing data and outliers resulted in a total of 2019 participants that were included as the final analysis sample (Fig. 1). No significant differences were found in the general characteristic between included and excluded participants.

### Ethics

This study was approved by the Institutional Review Board of Shanghai University of Sport (No. 102772020RT045). Permission to conduct the study was obtained from principals of each school. Signed informed consent forms were obtained from the children’s parents or guardians and from all participating children and adolescents prior to data collection. All methods in our study were performed in accordance with the guidelines and regulations of Declaration of Helsinki.

### Study variables

#### Sleep duration

Sleep duration was assessed through an open-ended question: “During the past month, how many hours of actual sleep did you get at night (This may be different from the number of hours you spent in bed)?” Participant responses on sleep duration were categorized into four groups as recommended by the NSF for children and adolescents [21, 33–35]: 1) very short sleep duration (i.e., sleep duratio*n* < 7 and < 6 h per night for children aged 6–13 years and adolescents aged 14–17 years, respectively); 2) short sleep duration (i.e., sleep duration of 7–8 and 6–7 h per night for children aged 6–13 years and adolescents aged 14–17 years, respectively); 3) recommended sleep duration (i.e., sleep duration of 9–11 and 8–10 h per night for children aged 6–13 years and adolescents aged 14–17 years, respectively); and 4) long sleep duration (i.e., sleep duratio*n* > 11 and > 10 h per night for children aged 6–13 years and adolescents aged 14–17 years, respectively).

#### Sleep quality

Sleep quality was measured with a Chinese version of the Pittsburgh Sleep Quality Index questionnaire (PSQI) [36, 37], which was confirmed to be a reliable and valid instrument to measure the sleep quality of Chinese children’s sleep quality [37]. This instrument comprised 18 items grouped into the following seven components: subjective sleep quality (very good, good, bad, very bad), sleep latency (< 15, 16–30, 31–60, and > 60 min), sleep duration (> 7, 6–7, 5–6, and < 5 h), habitual sleep efficiency (> 85%, 75–84%, 65–74%, and < 65%), sleep disturbances (0, 1–9, 10–18, and 19–27), use of sleep medication (none during the past month, < 1 time per week, 1–2 times per week, and > 3 times per week), and daytime dysfunction (0, 1–2, 3–4, and 5–6). Each component score was weighted equally from 0 (no difficulty) to 3 (severe difficulty) points. The total scores of the seven components were then summed to yield a global PSQI score ranging from 0–21 points; high global scores indicate poor subjective sleep quality. The global score was dichotomized into > 5 and ≤ 5 to evaluate the poor or good sleep quality, respectively [36].

#### Anthropometric measurements

Anthropometric measurements included children and adolescent body height (m) and body weight (kg). The height and weight of children and adolescents were measured by a portable instrument (GMCS-IV; Jianmin, Beijing, China) possibly with minimum clothing and without shoes. The weight was measured to the nearest 0.1 kg, and the height was measured to the nearest 0.01 m. Their body mass index (BMI) was calculated as weight in kilograms divided by height in meters square (kg/m^2^). All anthropometric values comprised the mean of three measures. The weight status of children and adolescents in this study were classified into two groups of overweight/obesity and normal weight based on the age- and sex-specific BMI cutoffs in the “Screening for overweight and obesity among school-age children and adolescents” (WS/T 586–2018) [38, 39], which is developed by National Health Commission of the People’s Republic of China (2018). These criteria apply to children and adolescents aged 6–18 years and provide the age- and sex-specific BMI cut-offs with units of 0.5 years (Additional file 1). Moreover, these cut-offs were widely used in a group of studies to determine the weight status of children and adolescents in China [39–41].

#### Covariates

Moderate-to-vigorous physical activity (MVPA), sedentary behavior, and demographic information, such as sex, age, and education level of parents were included as covariates because they were reported to influence weight status and sleep of children and adolescents [16, 42]. MVPA was assessed using the validated Chinese version of the International Physical Activity Questionnaire-Short Form (IPAQ-SF) [43]. The IPAQ-SF had been widely used in studies to measure PA level of children and adolescents in China [44, 45]. It includes three items and investigates frequency (times/week) and duration (mins/time) of walking, moderate-, and vigorous-intensity PA in past seven days. Time spent on MVPA each day was calculated as a sum of the minutes of moderate- and vigorous-intensity activities for at least 10 min in duration. Sedentary behavior was assessed using the Chinese version of Adolescent Sedentary Activity Questionnaire (ASAQ) [46], which was used to assess time spent on 14 different sedentary behaviors before and after school on each day of the week and on each day of the weekend. Time spent on each sedentary activity was calculated and summed to yield the total time per week spent in sedentary behavior. Demographic information, including participant’s sex, age, school levels (primary and middle schools), and education level of parents (less than high school, high school, bachelor’s degree, and master’s degree and above) was also obtained from participant responses to the survey.

### Data collection

Data were collected by the first and third authors in regular physical education classes (range: 35–40 min). This study provided explanations to participants before survey administration. All participants were given instructions and directed to complete the questionnaires. Questionnaires could be completed for approximately 15 min and were immediately collected upon completion. Weight (kg) and height (m) for each participant were measured after the survey.

### Statistical analysis

All analyses were performed with the Statistical Package for Social Science for Mac, version 24 (IBM Crop., Armonk, NY, USA). All continuous variables were distributed normally by the Kolmogorov–Smirnov test and the Shapiro–Wilk test (*p* < 0.001). Descriptive statistics were calculated for continuous variables with means and standard deviations and categorical variables with proportion. The differences in the continuous and categorical variables were analyzed using t-test and Chi-square (X^2^) test, respectively. Pearson and Spearman correlations were conducted to determine if children and adolescents’ sleep quality, sleep duration, covariates, and weight status were correlated. A series of multivariate logistic regression analysis was performed to estimate the odd ratios (ORs) and 95% confidence intervals (CIs) of weight status which was categorized as normal weight (i.e., reference group) and overweight/obesity by sleep quality (i.e., sleep quality scores) and sleep duration which was categorized as “very short sleep duration”, “short sleep duration”, “recommended sleep duration”, and “long sleep duration”. All analyses were performed separately with boys and girls and with children and adolescents to identify sex and age differences in results. All the tests were two-sided, and the significance level was set at 0.05.

## Results

### Descriptive characteristics of participants

The mean age was 11.56 years (SD = 1.84) ranging from 8 to16 years, and 50.97% were boys. According to the age classification of NSF, nearly three quarters (74.39%) of the participants were children (8–13 years old), and the others (25.61%) were adolescents (14–16 years old). The mean BMI of total participants was 19.42 kg/m^2^ and the prevalence of overweight/obesity was 30.31%. The mean sleep duration is 8.21 h (SD = 1.25). Approximately 5.89%, 51.26%, 42.15%, and 0.69% of the participants had very short sleep, short sleep, recommended sleep, and long sleep durations, respectively. Moreover, 79.35% of the participants reported good sleep quality and 20.65% reported poor sleep quality in the past month. The descriptive characteristics of the participants stratified by sex and age group are presented in Table 1.

**Table 1:** Participant characteristics stratified by sex and age group

Variable	Total	Boys	Girls	P	Children aged 8–13 years	Adolescents aged 14–16 years	p
	**N = 2019**	***n *= 1029**	***n *= 990**		***n *= 1502**	***n *= 517**	
**Age, years [M (SD)]**	11.56 (1.84)	11.67 (1.90)	11.59 (1.82)	0.374	10.79 (1.32)	14.08 (0.66)	** < 0.001**
**School level n (%)**				0.283			** < 0.001**
Primary School	899 (44.53)	446 (43.34)	453 (45.76)		899 (59.85)	0	
Middle school	1120 (55.47)	583 (56.66)	537 (54.24)		603 (40.15)	517 (100)	
**Age group n (%)**							
Children aged 8–13 years	1502 (74.39)	756 (73.47)	746 (75.35)		\	\	
Adolescents aged 14–16 years	517 (25.61)	273 (26.53)	244 (24.65)		\	\	
**Sex**							0.333
Boys	1029 (50.97)	\	\		756 (50.33)	273 (52.80)	
Girls	990 (49.03)	\	\		746 (49.68)	244 (47.20)	
**Anthropometric Measurements**							
Height, m [M (SD)]	1.53 (0.13)	1.55 (0.10)	1.51 (0.11)	** < 0.001**	1.48 (0.11)	1.66 (0.08)	** < 0.001**
Weight, kg [M (SD)]	46.32 (13.75)	48.96 (14.78)	43.58 (12.00)	** < 0.001**	42.36 (11.94)	57.83 (12.09)	** < 0.001**
BMI, kg/m^2^ [M (SD)]	19.42 (3.69)	20.07 (3.81)	18.75 (3.43)	** < 0.001**	18.94 (3.60)	20.82 (3.60)	** < 0.001**
**Weight Status n (%)**				** < 0.001**			**0.023**
Normal weight	1407 (69.69)	639 (31.65)	768 (38.04)		1026 (50.82)	136 (6.74)	
Overweight/obesity	612 (30.31)	390 (19.32)	222 (11.00)		476 (23.58)	381 (18.87)	
**Father Education n (%)**				0.108			** < 0.001**
< High school	153 (7.58)	90 (8.75)	63 (6.63)		135 (8.99)	18 (3.48)	
High school	297 (14.71)	152 (14.77)	145 (14.65)		221 (14.71)	76 (14.70)	
Bachelor’s degree	1195 (59.19)	611 (59.38)	584 (58.99)		814 (54.19)	381 (73.69)	
Master’ degree	374 (18.52)	176 (17.10)	198 (20.00)		332 (22.10)	42 (8.12)	
**Mother Education n (%)**				0.218			** < 0.001**
< High school	174 (8.62)	100 (9.72)	74 (7.47)		143 (9.52)	31 (6.00)	
High school	266 (13.17)	129 (12.54)	137 (13.84)		203 (13.52)	63 (12.19)	
Bachelor’s degree	1232 (60.97)	631 (61.32)	600 (60.60)		852 (56.72)	379 (73.3)	
Master’ degree	348 (17.24)	169 (16.42)	179 (18.08)		304 (20.24)	44 (8.51)	
**MVPA, min [M (SD)]**	65.70 (62.84)	72.01 (65.42)	59.13 (59.38)	** < 0.001**	66.61 (63.50)	63.05 (60.89)	0.257
**Sedentary Behavior, min [M (SD)]**	348.64 (175.10)	355.84 (185.25)	367.61 (177.26)	0.142	350.64 (177.72)	393.47 (183.23)	** < 0.001**
**Sleep Quality [M (SD)]**	3.00 (2.73)	3.40 (2.67)	3.69 (2.77)	**0.018**	3.38 (2.64)	4.02 (2.90)	**0.003**
Good sleep quality n (%)	1602 (79.35)	833 (80.95)	769 (77.68)		1216 (81.96)	386 (74.66)	
Poor sleep quality n (%)	417 (20.65)	196 (19.05)	221 (22.32)		286 (19.04)	131 (25.34)	
**Sleep Duration, h [M (SD)]**	8.21 (1.25)	8.23 (1.26)	8.17 (1.25)	0.307	8.85 (1.17)	7.34 (1.07)	** < 0.001**
Very short sleep duration n (%)	119 (5.89)	60 (5.83)	59 (5.96)		61 (4.06)	58 (11.22)	
Short sleep duration n (%)	1035 (51.26)	503 (48.89)	532 (53.74)		704 (46.87)	331 (64.02)	
Recommended sleep duration n (%)	851 (42.15)	457 (44.41)	394 (39.80)		723 (48.14)	128 (24.75)	
Long sleep duration n (%)	14 (0.69)	9 (0.87)	5 (0.51)		14 (0.93)	0	

*Note*: *M* mean, *SD* standard deviation, *BMI* body mass index, *MVPA* moderate to vigorous physical activity; *p* values were calculated by Chi-square (X^2^) test for categorical variables and t-tests for continuous variables between sex and school level, and p values with bold type indicated *p* < 0.05

Table 2 presented the bivariate Pearson or Spearman correlations between all study variables in this study. All study variables were significantly correlated except for the following non-significant relationships. Weight status was not correlated with father and mother educational level, MVPA, and sleep duration and quality. School level was not significantly correlated with sex and MVPA. Age was also not correlated with sex.

**Table 2:** Intercorrelations for all study variables (*n *= 2005)^b^

Variable	Weight status	Sex	Father educational level	Mother educational level	Age	MVPA	SED	Sleep duration
Weight status	\							
Sex	-0.17^**^	\						
Father educational level	-0.04	0.05^*^	\					
Mother educational level	-0.01	0.03	0.71^**^	\				
Age	-0.07^**^	-0.02	-0.09^**^	-0.07^**^	\			
MVPA	0.01	-0.11^**^	-0.09^**^	-0.08^**^	-0.06^**a^	\		
SED	-0.02	0.05^*^	-0.05^*^	-0.07^**^	0.14^**a^	0.09^**a^	\	
Sleep duration	-0.03	-0.05^*^	0.01	-0.01	-0.34^**^	0.05^*^	-0.13^**^	\
Sleep quality	0.02	0.06^*^	-0.04	-0.03	0.13^**a^	-0.08^**a^	0.19^**a^	-0.34^**^

Note: *p < 0.01, **p < 0.05. ^a^Pearson correlation coefficient. ^b^Without participants with long sleep duration

### Sex and age group difference in sleep duration, sleep quality, and weight status

Sex and age group differences in sleep duration, sleep quality, and weight status are presented in Table 1. Significant sex (X^2^ = 0.94, df = 1, *p* < 0.001) and age group differences (X^2^ = 5.28, df = 1, *p* = 0.023) were found in weight status, with higher overweight/obesity prevalence among boys (19.32%) and children (23.58%) than girls (11.00%) and adolescents (18.87%), respectively. Girls (M = 3.69, SD = 2.77) and adolescents (M = 4.02, SD = 2.90) had significantly higher sleep quality scores than boys (M = 3.40, SD = 2.67) and children (M = 3.38, SD = 2.64) (t = -2.36, *p* = 0.018; t = -4.38, *p* < 0.001). Significant age group difference in sleep duration was also observed (t = 21.72, *p* < 0.001), with longer sleep duration in children (M = 8.85, SD = 1.17) than adolescents (M = 7.34, SD = 1.07). No significant sex differences were observed in sleep duration (t = 1.02, *p* = 0.307).

#### Association between sleep quality, sleep duration, and overweight/obesity

The association between sleep duration, sleep quality, and overweight/obesity was analyzed (Table 3). The group of samples with long sleep duration was not included in regression model because of small sample size (*n* = 14) and low prevalence of long sleep duration (0.69%) and thus could not provide sufficient statistical power [47, 48]. Univariate associations between sleep duration, sleep quality, and overweight/obesity of all participants were initially analyzed in Model 1. No significant relationships between sleep duration, sleep quality, and overweight/obesity were found. Model 2 showed that short sleep duration was more likely to be overweight/obesity (OR = 1.34, 95% CI: 1. 08–1.66) than recommended sleep duration. This finding was obtained after adjusting for covariates. However, no significant relationship between sleep quality and overweight/obesity was found in Model 2. Further analyses were conducted to determine the association of sleep duration with overweight/obesity independent of sleep quality. Accordingly, sleep duration and quality were simultaneously entered into Model 3 with covariates. The analytical results showed that short sleep duration was still associated with a high risk of overweight/obesity (OR = 1.32, 95%CI: 1.06–1.64). This finding suggested that the association of short sleep duration with overweight/obesity was independent of sleep quality. The relationship between sleep quality and overweight/obesity remains insignificant.

**Table 3:** Unadjusted and adjusted logistic regression analysis of sleep quality and sleep duration associated with overweight/obesity among total sample and by sex and age

Variables	Total		Boys		Girls		Children aged 8–13 years				Adolescents aged 14–16 years		
	**OR (95%CI)**	**p**	**OR (95%CI)**	**p**	**OR (95%CI)**	**p**	**OR (95%CI)**	**p**			**OR (95%CI)**		**p**
**Univariable model 1**													
**Sleep Quality Score**	1.02 (0.98 to 1.05)	0.351	0.99 (0.94 to 1.04)	0.621	**1.07 (1.02 to 1.13)**	**0.010**	1.03 (0.99 to 1.07)		0.190		1.00 (0.94 to 1.07)		0.921
**Sleep Duration**													
Very short sleep duration	1.21 (0.80 to 1.82)	0.372	1.15 (0.66 to 1.99)	0.626	1.40 (0.74 to 2.65)	0.297	1.53 (0.90 to 2.62)		0.120		1.14 (0.56 to 2.63)		0.721
Short sleep duration	1.12 (0.92 to 1.37)	0.255	1.08 (0.83 to 1.40)	0.564	1.32 (0.96 to 1.81)	0.089	1.16 (0.93 to 1.45)		0.190		1.24 (0.77 to 1.99)		0.377
Recommended sleep duration^a^	1		1		1		1				1		
**Multivariate model 2**													
**Age**	**0.91 (0.86 to 0.96)**	**0.001**	**0.86 (0.80 to 0.93)**	** < 0.001**	0.98 (0.89 to 1.07)	0.626	**0.91 (0.83 to 0.99)**		**0.033**	0.82 (0.60 to 1.12)		0.201	
**Sex**													
Boys	**2.18 (1.79 to 2.66)**	** < 0.001**	\		\		**2.51 (2.00 to 3.15)**		** < 0.001**	1.34 (0.88 to 2.04)		0.180	
Girls ^a^	1	1	\		\		1			1			
**Father Education**													
Less than high school	1.29 (0.74 to 2.26)	0.365	1.72 (0.82 to 3.60)	0.152	0.90 (0.38 to 2.14)	0.803	1.27 (0.69 to 2.35)		0.444	1.97 (0.45 to 8.53)		0.366	
High school	1.57 (0.97 to 2.54)	0.068	1.95 (1.00 to 3.78)	0.05	1.20 (0.59 to 2.44)	0.626	1.64 (0.96 to 2.80)		0.070	1.14 (0.34 to 3.81)		0.835	
Bachelor’s degree	1.24 (0.81 to 1.90)	0.319	1.45 (0.81 to 2.59)	0.215	1.05 (0.56 to 1.96)	0.891	1.26 (0.79 to 2.00)		0.328	1.03 (0.34 to 3.14)		0.964	
Master’ degree ^a^	1	1	1		1		1			1			
**Mother Education**													
Less than high school	0.95 (0.55 to 1.63)	0.839	0.71 (0.34 to 1.48)	0.358	1.50 (0.67 to 3.38)	0.326	0.89 (0.48 to 1.64)		0.710	1.28 (0.35 to 4.75)		0.708	
High school	0.76 (0.46 to 1.26)	0.293	0.72 (0.36 to 1.43)	0.342	0.86 (0.42 to 1.79)	0.691	0.73 (0.42 to 1.27)		0.263	0.93 (0.28 to 3.08)		0.904	
Bachelor’s degree	0.79 (0.52 to 1.22)	0.289	1.02 (0.57 to 1.82)	0.955	0.58 (0.31 to 1.10)	0.093	0.84 (0.53 to 1.34)		0.469	0.70 (0.23 to 2.08)		0.519	
Master’ degree ^a^	1	1	1		1		1			1			
**MVPA**	**1.00 (1.00 to 1.00)**	**0.037**	**1.00 (1.00 to 1.00)**	**0.005**	1.00 (1.00 to 1.00)	0.743	1.00 (1.00 to 1.00)		0.135	1.00 (0.99 to 1.00)		0.188	
**Sedentary Behavior**	1.00 (1.00 to 1.00)	0.928	1.00 (1.00 to 1.00)	0.705	1.00 (1.00 to 1.00)	0.619	1.00 (1.00 to 1.00)		0.784	1.00 (1.00 to 1.00)		0.529	
**Sleep Quality Score**	1.03 (0.99 to 1.07)	0.146	0.99 (0.94 to 1.04)	0.595	**1.08 (1.02 to 1.14)**	**0.009**	1.03 (0.99 to 1.07)		0.196	1.01 (0.94 to 1.09)		0.781	
**Age**	**0.89 (0.84 to 0.95)**	** < 0.001**	**0.85 (0.78 to 0.91)**	** < 0.001**	0.97 (0.88 to 1.06)	0.468	0.88 (0.80 to 0.97)		0.007	0.83 (0.61 to 1.14)		0.245	
**Sex**													
Boys	**2.20 (1.80 to 2.69)**	** < 0.001**	\		\		**2.54 (2.02 to 3.20)**		** < 0.001**	**1.34 (0.88 to 2.04)**		**0.168**	
Girls ^a^	1		\		\		1			1			
**Father Education**													
Less than high school	1.30 (0.74 to 2.27)	0.365	1.67 (0.80 to 3.49)	0.176	0.96 (0.40 to 2.31)	0.93	1.26 (0.68 to 2.33)		0.466	1.99 (0.46 to 8.60)		0.358	
High school	1.57 (0.97 to 2.54)	0.07	1.90 (0.98 to 3.68)	0.059	1.22 (0.60 to 2.51)	0.583	1.62 (0.95 to 2.77)		0.079	1.16 (0.35 to 3.90)		0.811	
Bachelor’s degree	1.24 (0.81 to 1.89)	0.328	1.43 (0.80 to 2.56)	0.224	1.03 (0.55 to 1.95)	0.919	1.25 (0.79 to 1.99)		0.341	1.05 (0.34 to 3.21)		0.933	
Master’ degree ^a^	1		1		1		1			1			
**Mother Education**													
Less than high school	0.96 (0.56 to 1.67)	0.893	0.73 (0.35 to 1.54)	0.411	1.43 (0.63 to 3.25)	0.387	0.93 (0.50 to 1.71)		0.807	1.25 (0.34 to 4.64)		0.738	
High school	0.78 (0.47 to 1.28)	0.325	0.74 (0.37 to 1.47)	0.388	0.84 (0.41 to 1.76)	0.651	0.76 (0.43 to 1.33)		0.328	1.90 (0.27 to 3.00)		0.862	
Bachelor’s degree	0.79 (0.51 to 1.21)	0.277	1.03 (0.58 to 1.84)	0.927	0.57 (0.30 to 1.07)	0.082	0.84 (0.53 to 1.35)		0.476	0.68 (0.23 to 2.04)		0.495	
Master’ degree ^a^	1		1		1		1			1			
**MVPA**	**1.00 (1.00 to 1.00)**	**0.037**	**1.00 (1.00 to 1.00)**	**0.006**	1.00 (1.00 to 1.00)	0.741	1.00 (1.00 to 1.00)		0.135	1.00 (0.99 to 1.00)		0.177	
**Sedentary Behavior**	1.00 (1.00 to 1.00)	0.867	1.00 (1.00 to 1.00)	0.568	1.00 (1.00 to 1.00)	0.382	1.00 (1.00 to 1.00)		0.738	1.00 (1.00 to 1.00)		0.649	
**Sleep Duration**													
Very short sleep duration	1.49 (0.97 to 2.29)	0.071	1.39 (0.78 to 2.46)	0.261	1.53 (0.78 to 3.01)	0.217	1.68 (0.96 to 2.93)		0.071	1.15 (0.55 to 2.41)		0.710	
Short sleep duration	**1.34 (1.08 to 1.66)**	**0.008**	1.25 (0.94 to 1.64)	0.123	**1.51 (1.06 to 2.13)**	**0.021**	**1.34 (1.05 to 1.71)**		**0.019**	**1.22 (0.75 to 1.98)**		**0.433**	
Recommended sleep duration ^a^	1		1		1		1			1			
**Multivariate logistic regression model 3**													
**Age**	**0.89 (0.84 to 0.95)**	** < 0.001**	**0.84 (0.78 to 0.91)**	** < 0.001**	0.96 (0.87 to 1.05)	0.345	**0.88 (0.80 to 0.97)**		**0.007**	0.83 (0.60 to 1.14)		0.242	
**Sex**													
Boys	**2.21 (1.81 to 2.69)**	** < 0.001**	\		\		**2.54 (2.02 to 3.20)**		** < 0.001**	1.35 (1.88 to 2.06)		0.166	
Girls ^a^	1		\		\		1			1			
**Father Education**													
Less than high school	1.29 (0.74 to 2.26)	0.375	1.69 (0.81 to 3.56)	0.165	0.92 (0.38 to 2.21)	0.847	1.26 (0.68 to 2.32)		0.47	1.97 (0.45 to 8.55)		0.365	
High school	1.56 (0.96 to 2.53)	0.071	1.92 (0.99 to 3.74)	0.055	1.21 (0.59 to 2.47)	0.608	1.62 (0.95 to 2.77)		0.079	1.15 (0.34 to 3.88)		0.818	
Bachelor’s degree	1.23 (0.81 to 1.89)	0.333	1.45 (0.81 to 2.60)	0.212	1.03 (0.55 to 1.94)	0.924	1.25 (0.79 to 1.99)		0.346	1.05 (0.34 to 3.20)		0.937	
Master’ degree ^a^	1		1		1		1			1			
**Mother Education**													
Less than high school	0.96 (0.56 to 1.66)	0.887	0.74 (0.35 to 1.55)	0.424	1.47 (0.65 to 3.32)	0.358	0.92 (0.50 to 1.71)		0.8	1.25 (0.34 to 4.64)		0.737	
High school	0.78 (0.47 to 1.29)	0.326	0.74 (0.37 to 1.47)	0.387	0.86 (0.41 to 1.78)	0.678	0.76 (0.43 to 1.32)		0.327	0.90 (0.27 to 2.99)		0.862	
Bachelor’s degree	0.78 (0.52 to 1.21)	0.284	1.02 (0.57 to 1.82)	0.96	0.57 (0.30 to 1.08)	0.087	0.85 (0.53 to 1.35)		0.483	0.68 (0.23 to 2.04)		0.494	
Master’ degree ^a^	1		1		1		1			1			
**MVPA**	**1.00 (1.00 to 1.00)**	**0.041**	**1.00 (1.00 to 1.00)**	**0.005**	1.00 (1.00 to 1.00)	0.647	1.00 (1.00 to 1.00)		0.141	1.00 (1.00 to 1.00)		0.188	
**Sedentary Behavior**	1.00 (1.00 to 1.00)	0.946	1.00 (1.00 to 1.00)	0.703	1.00 (1.00 to 1.00)	0.587	1.00 (1.00 to 1.00)		0.786	1.00 (1.00 to 1.00)		0.528	
**Sleep Quality Score**	1.01 (0.97 to 1.06)	0.533	0.97 (0.92 to 1.02)	0.247	**1.07 (1.01 to 1.14)**	**0.028**	1.01 (0.96 to 1.06)		0.677	1.01 (0.93 to 1.09)		0.884	
**Sleep Duration**													
Very short sleep duration	1.40 (0.87 to 2.25)	0.167	1.61 (0.86 to 3.02)	0.134	1.07 (0.50 to 2.26)	0.870	1.59 (0.87 to 2.93)		0.295	1.12 (0.48 to 2.59)		0.195	
Short sleep duration	**1.32 (1.06 to 1.64)**	**0.013**	1.28 (0.97 to 1.70)	0.084	1.38 (0.97 to 1.98)	0.075	**1.33 (1.03 to 1.70)**		**0.027**	1.21 (0.73 to 1.99)		0.463	
Recommended sleep duration ^a^	1		1		1		1			1			

*Note:* ^a^: Reference Category; *OR* odd ratio, *CI* confidence interval; The values of *p* < 0.05 were highlighted in bold

Univariable Model 1: Univariate association of sleep quality and sleep duration and overweight/obesity

Multivariable Model 2: Sleep quality and sleep duration separately entered into multivariable model after adjusting for the participants' sex, age, parental education, moderate to vigorous physical activity duration per day, and sedentary behavior duration per day

Multivariable Model 3: Sleep quality and sleep duration were simultaneously entered into multivariable model after adjusting for the participants' sex, age, parental education, moderate to vigorous physical activity duration per day, and sedentary behavior duration per day

Regressions were performed for boys and girls separately to understand sex differences in sleep–obesity relationship. Significant relationships between sleep quality and overweight/obesity were found for girls in Models 1–3 (OR = 1.07, 95% CI: 1.02–1.13; OR = 1.08, 95% CI: 1.02–1.14; OR = 1.07, 95% CI: 1.01–1.14). Short sleep duration was more likely to be overweight/obesity than recommended sleep duration (OR = 1.51, 95% CI: 1. 06–2.13) after adjusting for covariates in Model 2. However, the association of short sleep duration and overweight/obesity became insignificant when sleep duration, sleep quality, and covariates simultaneously were entered into Model 3. For boys, no significant relationships between sleep quality, sleep duration, and overweight/obesity were found in Models 1–3 (Table 3).

Regressions were performed for children and adolescents to explore age group differences in sleep–obesity relationship. Results showed that short sleep duration significantly increased risk of overweight/obesity (OR = 1.34, 95% CI: 1.05–1.71) among children after adjusting for covariates in Model 2. The association of short sleep duration and overweight/obesity remained significant (OR = 1.33, 95% CI: 1.03–1.70) when sleep duration, sleep quality, and covariates were simultaneously entered into Model 3. The relationship between sleep quality and obesity was not found among children. For adolescents, no significant relationships between sleep quality, sleep duration, and overweight/obesity were found in Models 1–3 (Table 3).

## Discussion

Descriptive results showed that 30.31% of children and adolescents were overweight or obese. This value is considerably higher than the global prevalence of overweight and obesity in children and adolescents with 12.9% in girls and 13.4% in boys [1]. This finding confirmed the fact that overweight/obesity is one of the important health threats for Chinese children and adolescents. Survey results also indicated that more than half of the participants (57.15%) do not reach the sleep duration recommendation by NSF [35] in the current study. The result is consistent with previous Chinese studies, in which insufficient sleep prevalence ranges from 31.3% to 66.0% [15, 19, 49], and higher than those studies from Western countries with 14.2% to 50.0% prevalence [16, 50, 51]. The differences in the prevalence of insufficient sleep may be related to the unique culture and educational system in China, where education is based on Confucian principles addressing the successful scholarship of children and adolescents [52]. Influenced by the Confucian principles, parents and teachers place particular emphasis on children and adolescent academic performance. Most Chinese children and adolescents must spend an increasing amount of time studying and attending some kind of private classes at night at the “expense” of sleep time [53]. Additionally, the results on the sleep quality of children and adolescents are optimistic, and 79.35% of participants reported good sleep quality. This finding is aligned with previous international and Chinese local studies [16, 51, 54] and reasonable because sleep disorders do not frequently occur till the middle and old age [55].

The present study investigated the relationship between sleep quality and duration with overweight/obesity of children and adolescents in China. The research results indicated that short sleep duration (7–8 and 6–7 h per night for children aged 6–13 years and adolescents aged 14–17 years) increased the likelihood of overweight/obesity for children and adolescents compared with recommended sleep duration (9–11 h and 8–10 h per night for children aged 6–13 years and adolescents aged 14–17 years). This finding is consistent with previous studies [56, 57] and confirmed a finding from a systematic review by Wu et al.[20]. Several potential mechanisms, such as hormonal change, increased energy intake and decreased energy consumption, and reduced metabolic rate might explain the association between short sleep and increased odds of overweight/obesity. First, insufficient sleep may affect hormonal levels, such as decreasing leptin and increasing ghrelin. The changes in the hormonal levels may increase appetite, thereby leading to increased weight gain and overweight/obesity [58]. Second, epidemiological evidence showed that insufficient sleep may lead to tiredness, which is related to reduce PA participation and increased sedentary time [59]. Moreover, sleep loss may increase dietary intake due to additional waking hours available for eating [60]. Decreased energy consumption and increased energy intake promoted weight-gain among children and adolescents. Third, reduced sleep may reduce the basal metabolic rate [61]. Thus, excess calories can easily be converted into fat accumulation in the body after eating, which also introduces additional weight for children and adolescents. However, no significant relationship between very short sleep duration (< 7 and < 6 h for 8–13 and 14–16 years old, respectively) and overweight/obesity among the children and adolescent samples in this study. This finding is different from previous studies [62]. The group reporting very short sleep duration was rare (*n* = 119, 5.89%) in this study and the limited sample size could not provide sufficient statistical power to detect such effects on weight status. Further studies with a larger sample size are necessary to examine the relationship between sleep and weight status [56].

However, findings indicated that sleep quality is insignificantly related to obesity among children and in this study, which is in agreement with the findings of previous studies [17, 18, 51]. Moreover, research results showed that short sleep duration was related to obesity, independent of sleep quality. The two findings confirmed that the major contributor of weight status among participants in this study is short sleep duration rather than sleep quality. The cause of the nonsignificant relationship between sleep quality and weight status remains unclear. In the present study, it is possible that sleep quality of most participants in the present is good and cannot explain the high overweight/obesity prevalence. These findings are important for the health of children and adolescents under Chinese culture. On the one hand, Chinese people traditionally believe that long sleep duration leads to obesity. Thus, parents do not place emphasis on the sleep of their children [63]. This finding may provide new insights into the cognition of Chinese people on the sleep-obesity relationship. On the other hand, the present study and previous Chinese local studies [64–66] all showed that sleep loss is serious among children and adolescents in China due to considerable educational pressure and emphasis on educational excellence. Therefore, some effective interventions to extend sleep duration, such as earlier bedtime and later school start time, must be encouraged in the prevention and treatment of children and adolescents’ obesity [67].

Sex differences exist in the relationship between sleep duration, and weight status among children and adolescents in China. Short sleep duration only increased the risk for overweight/obesity of girls after multivariable adjustment, but not for boys. This finding is in agreement with some previous Chinese studies [49, 57]. Sex differences in the physiology in puberty may explain this finding. During the period of puberty, muscle mass is increased and fat mass is reduced in boys, whereas fat mass is increased in girls [19]. Moreover, the basic metabolic rate of boys is higher than girls, thus increasing energy expenditure of boys [26]. These conditions could all be protective factors for overweight and obesity of boys, thus boys are less susceptible to short sleep after sleep reduction than girls. However, the opposite findings from some studies conducted in Western and Middle Eastern countries revealed that reduced sleep is more related to obesity in boys than girls [31]. The difference may be attributed to unique sex differences in academic learning in China. Girls are reported to study harder than boys to obtain academic achievements in primary and middle schools in China [20]. Girls may spend additional time studying out of schools, which may later result in less sleep than boys. The descriptive results in this study also confirmed that the prevalence of short and very short sleep among girls is higher than boys. This finding shows further influence on weight status among girls. Moreover, sleep quality increased the risk of overweight/obesity of girls instead of boys, which was consistent with previous studies [50, 68, 69]. Some researchers found that poor sleep quality was more likely to trigger higher level of triglycerides of girls than boys, which increase serum lipids level and may result in overweight/obesity [22]. Thus, possible interventions tailored to different sex are necessary for China. In particular, additional efforts are required to assure sufficient sleep and good sleep quality of girls. Although short sleep duration and sleep quality were associated with increased risks of overweight/obesity among girls instead of boys, the obesity prevalence of boys is significantly higher than girls in the present study. Except for sleep, the adiposity of children and adolescents is influenced by some other factors, such as behavioral and environmental factors. These factors possibly contributed more to the obesity of boys than girls. Future studies are needed to explore the sex differences in the association between these factors and the obesity of children and adolescents.

Findings also showed that age difference existed in the relationship between sleep duration and weight status of children and adolescents. Short sleep duration was associated with overweight/obesity of children, independent of sleep quality, whereas no significant relationship existed in adolescents. This finding was confirmed by a previous systematic review [24], which found that short sleep duration is associated with higher risk of overweight/obesity of children aged less than 10 years, but the association is somewhat inconsistent among adolescents aged more than 10 years. Perhaps the stronger association of short sleep duration with BMI among children compared to adolescents is partially due to pubertal development. Puberty is accompanied by a higher basal metabolic rate and increased muscle mass, which decreased BMI and thus mitigated the negative effect of short sleep on BMI [28, 29]. Another potential reason may be the smaller sample size of adolescents (*n* = 517) than children (*n* = 1502). A larger sample size may increase the probability of detecting statistical significance of a specific effect [70, 71]. Given the evidence that the association between sleep duration and obesity may differ from age, the effect of age should be considered carefully when drawing any conclusions about short sleep duration as a risk factor for obesity [28].

### Strength and limitation

The authors believe that this work is one of the few studies to examine the sex and age difference in association between sleep duration, sleep quality, and weight status of children and adolescents in China. The findings of this study would help provide interventions to improve weight status among Chinese children and adolescents. Nevertheless, the limitations inherent in the current study should be noted. The first limitation is the generalization of the results. The participants comprised students from six primary and middle schools in Shanghai, thus failing to represent the children and adolescents from other schools and areas of China completely. Future studies may expand the research scope by employing a large and diverse sample (e.g., samples covering additional schools, grade levels, and other cities in China). Second, the cross-sectional data preclude any causal inference on the relationships between sleep and weight status. Longitudinal research is needed to establish any sort of causal relationship between the two factors. Third, sleep duration and quality were measured on the basis of self-reports, which are known to produce recalls or response biases. In addition, we measured sleep duration per night instead of per day, which may bring results bias and underestimate the true sleep duration for children and adolescents in China. Using additional objective sleep measures (e.g., accelerometer) to measure nap and sleep duration at night may increase the predictive power of sleep because of low levels of measurement error. Fourth, the sleep duration during weekend days and weekdays was not distinguished, thus possibly introducing inaccuracy for the data of sleep duration and quality. Finally, five covariates, including age, sex, parental education, MVPA, and sedentary behavior, were adjusted in this study. Other covariates (e.g., dietary habits, anxiety and depression, and neighborhood and family context) should be considered in future studies.

## Conclusion

Overall, short sleep duration is associated with increased risk of overweight/obesity of children and adolescents in China, independent of sleep quality. This relationship is significant for children instead of adolescents. Short sleep duration and sleep quality were significantly associated overweight/obesity in girls but not boys in China. Based on the high prevalence of insufficient sleep among children and adolescents in China, interventions to extend sleep duration of children and adolescents are needed. Additional emphasis may be placed on girls to ensure their sufficient sleep and good sleep quality.

## Supplementary Information

**Additional file 1:** Age- and sex-specific body mass index cutoff points for Chinese children and adolescents.

**Additional file 2:** Informed consent forms.

**Additional file 3: **

## Data Availability

The datasets generated and/or analysed during the current study are available in “Additional file 3” of the submission.

## Abbreviations

BMI: Body Mass Index
CNSSCH: Chinese National Survey on Students’ Constitution and Health
NSF: The U.S. National Sleep Foundation
PSQI: Pittsburgh Sleep Quality Index questionnaire
MVPA: Moderate-to-Vigorous Physical Activity
IPAQ-SF: International Physical Activity Questionnaire-Short Form
ASAQ: Adolescent Sedentary Activity Questionnaire
CI: Confidence Interval
OR: Odd Ratio
M: Mean
SD: Standard Deviation
