# Supplementary material for: Associations between sleep duration, sleep quality, and weight status in Chinese children and adolescents
Source: BMC Public Health. 2022 Jun 7;22:1136. doi: 10.1186/s12889-022-13534-w (PMC9172025; doi:10.1186/s12889-022-13534-w)
Supplement: Supplementary file 1 — Additional file 1: Age- and sex-specific body mass index cutoff points for Chinese children and adolescents. [file 12889_2022_13534_MOESM1_ESM.pdf]

Additional file 1: Age- and sex-specific body mass index (BMI) cutoff points for Chinese children and adolescents

Age (years)	Male		Female	
	Overweight	Obesity	Overweight	Obesity
6.0~	16.4	17.4	16.2	17.5
6.5~	16.7	18.1	16.5	18.0
7.0~	17.0	18.7	16.8	18.5
7.5~	17.4	19.2	17.2	19.0
8.0~	17.8	19.7	17.6	19.4
8.5~	18.1	20.3	18.1	19.9
9.0~	18.5	20.8	18.5	20.4
9.5~	18.9	21.4	19.0	21.0
10.0~	19.2	21.9	19.5	21.5
10.5~	19.6	22.5	20.0	22.1
11.0~	19.9	23.0	20.5	22.7
11.5~	20.3	23.6	21.1	23.3
12.0~	20.7	24.1	21.5	23.9
12.5~	21.0	24.7	21.9	24.5
13.0~	21.4	25.7	22.6	25.0
13.5~	21.9	25.7	22.6	25.6
14.0~	22.3	26.1	22.8	25.9
14.5~	22.6	26.4	23.0	26.3
15.0~	22.9	26.6	23.2	26.6
15.5~	23.1	26.9	23.4	26.9
16.0~	23.3	27.1	23.6	27.1
16.5~	23.5	27.4	23.7	27.4
17.0~	23.7	27.6	23.8	27.6
17.5~	23.8	27.8	23.8	27.8
18.0	24.0	28.0	24.0	28.0
