# Supplementary material for: Associations between sleep duration, sleep quality, and weight status in Chinese children and adolescents
Source: BMC Public Health. 2022 Jun 7;22:1136. doi: 10.1186/s12889-022-13534-w (PMC9172025; doi:10.1186/s12889-022-13534-w)
Supplement: Supplementary file 2 — Additional file 2: Informed consent forms. [file 12889_2022_13534_MOESM2_ESM.pdf]

Additional file 2: Informed consent forms.

Informed consent forms

Dear Sir or Madam:

I will conduct a research project entitled "Associations between sleep duration, sleep quality, and weight status in Chinese children and adolescents". The purpose of this study is to examine the association between sleep and weight status of children and adolescents. I write to invite your children to participant in this research project.

This study is supervised by Professor Wang and is approved by the Institutional Review Board of Shanghai University of Sport (No. 102772020RT045). Your kids will complete the questionnaire in class and their height and weight will be measured as well. All data gathered during this research project will not require the names of you kids to be used and the data will be stored in confidence and anonymity.

During the periods of data collection, your children are free to withdraw from the study at any time without prejudice. If you have any queries about the captioned study, please contact me at 18207184217.

Thank you for your support in our research.

Yours sincerely,

____

School of Physical Education and Sport Training, SUS
Professor Li-Juan Wang

____

School of Physical Education and Sport Training, SUS
Huan Chen

…………………………………………………………………………………………

Parents or guardians Reply Form

My children____________________and I, have read the accompany description of the research project entitled conducted by Huan Chen, and will/will not (please delete as whichever inapplicable) consent to participate and will participant in this study. I understand that I may withdraw at anytime without prejudice.

Signature: ____________________

Date: ____________________
